# Evaluation of Acridine Orange Staining for a Semi-Automated Urinalysis Microscopic Examination at the Point-of-Care

**DOI:** 10.3390/diagnostics9030122

**Published:** 2019-09-18

**Authors:** Amy J. Powless, Sandra P. Prieto, Madison R. Gramling, Roxanna J. Conley, Gregory G. Holley, Timothy J. Muldoon

**Affiliations:** 1 Department of Biomedical Engineering, University of Arkansas, Fayetteville, AR 72701, USA; apowless@uark.edu (A.J.P.); spprieto@uark.edu (S.P.P.); 2 Pat Walker Health Center, University of Arkansas, Fayetteville, AR 72701, USA; gramling@uark.edu (M.R.G.); rjconley@uark.edu (R.J.C.); gholley@uark.edu (G.G.H.)

**Keywords:** urinalysis, urinary tract infection, acridine orange, fluorescence, point-of-care, cell classification

## Abstract

A urinary tract infection (UTI) can be diagnosed via urinalysis, consisting of a dipstick test and manual microscopic examination. Point-of-care (POC) image-based systems have been designed to automate the microscopic examination for low-volume laboratories or low-resource clinics. In this pilot study, acridine orange (AO) was evaluated as a fluorescence-based contrast agent to aid in detecting and enumerating urine sediment specific for diagnosing a UTI. Acridine orange staining of epithelial cells, leukocytes, and bacteria provided sufficient contrast to successfully implement image segmentation techniques, which enabled the extraction of classifiable morphologic features. Surface area bounded by each cell border was used to differentiate the sediment; epithelial cells were larger than 500μm^2^, bacteria were less than 30μm^2^, and leukocytes in between. This image-based semi-automated technique using AO resulted in similar cell counts to the clinical results, which demonstrates the feasibility of AO as an aid for POC urinalysis systems.

## 1. Introduction

Urinary tract infections (UTIs) are among the most common types of infection [1,2,3,4], and are caused by an overabundance of microorganisms, predominately Escherichia coli, in the urinary tract. However, fungal and yeast species can be the cause as well [4,5]. If left untreated, or treated improperly, the infection can lead to a more serious kidney infection. A urinalysis, consisting of a dipstick test and manual microscopic examination (MME), is performed to diagnose a UTI. It was the third most ordered laboratory test during office visits in the U.S. in 2015 [6]. A urinalysis is followed by a urine culture, when available, for diagnosis confirmation.

When a UTI is suspected, a dipstick test is the initial test performed in all laboratory settings ranging from small clinics to high-volume central laboratories. A positive result for leukocyte esterase or nitrate indicate the presence of leukocytes or bacteria, respectively [5,7], and an MME is then used to estimate the number of bacteria, leukocytes, and epithelial cells present in the specimen. This involves centrifugation of the specimen, resuspension of the sediment, and manual counts within high-powered fields in a brightfield or phase contrast microscope [4,7,8]. This process requires a trained laboratory technician and is time and labor intensive, and lack of standardization can lead to inconsistencies between technicians [9,10,11]. Dipstick tests and MMEs have shown varying accuracies, both individually and combined, with the majority of the studies demonstrating poor performance in diagnosing a UTI [12,13,14].

A urine culture is considered the gold standard for diagnosing a UTI and is performed when the dipstick test and MME yield positive results or when a patient has recurring UTIs [1,15]. Urine cultures can reveal the specific type of microorganism responsible for the infection to help guide treatment. However, a urine culture can take between 18–48 h to yield results [15,16,17]. Due to this delay, symptomatic patients are treated based on the dipstick test while waiting for results. Therefore, patients can be susceptible to under and over treatment, which could cause sepsis if treatment is delayed [18], or antibiotic resistance if the patient is given unnecessary treatment [15,19].

Automated urinalysis systems have been developed to improve test turnaround time and reduce interobserver error between technicians [8,20]. The Sysmex UF-1000i and UF-5000 are based on flow cytometry and have demonstrated good performance in rapidly screening for negative bacteria culture, although an MME is required for abnormal results [8,21,22]. The Iris iQ200, UriSed 3, and SediMax are digital microscopy-based systems that mimic the current MME [1,23,24]. Both acquire images of urine sediment either on a cell-by-cell basis or via wide-field view similar to the microscopic views technicians use to perform manual examinations [8]. Not only do these systems perform automated counts, they record images for re-evaluation by the technician, physician, or remote clinician. Automated urinalysis systems greatly benefit large, high-volume laboratories by quickly screening for negative results and reporting uncomplicated results that do not require additional tests, thus reducing the technicians’ workload [8,21]. In low-volume laboratories and clinics, these systems are unavailable due to costs exceeding US $40,000 and the requirement of extensive training [8,25]. Therefore, there is a need for rapid, automated systems that can perform a microscopic examination where well-equipped laboratories and experienced technicians are unavailable.

Point-of-care (POC) analyzers aim to address the inaccessibility of laboratory tests at or near a patient by designing systems that are portable, low-cost, and simple enough for an inexperienced user to operate [26]. Currently, there are many POC urine analyzers that focus on reading dipstick test strips to reduce observer bias in visually evaluating the color-based test strip against a color chart [27,28,29]. In general, these devices have good agreement with laboratory references standards; however, they continue to require an MME if there are positive results for nitrates or leukocyte esterase. Alternatively, Smith et al. have developed a low-cost cellphone-based system that incorporates both the dipstick test and microscopic examination capable of imaging epithelial cells and leukocytes. It has a total fabrication cost of $20 not including cellphone cost, and costs ~$1 per test [30]. Although the dipstick test was automated, the microscopic examination required trained users to interpret the images, and could not visualize bacteria. The differentiation of urine sediment based on cellular detail in microscopic images is a challenge to automate when using brightfield and phase contrast microscopy. Therefore, improvements in contrast, either optically or chemically, would enhance the segmentation and identification of urine sediment allowing for automated classification.

Acridine orange (AO) is a rapid, fluorescent vital dye known to stain live, nucleated cells and bacteria by preferentially staining DNA via intercalation and staining RNA via electrostatic interactions [31]. It has been used in POC blood analyzers to differentiate leukocytes [32,33,34], urinary tract cytology to detect bladder cancer [35,36], and microorganism detection in cerebrospinal fluid [37,38]. In different cellular environments, it emits unique fluorescence ranging from 530 nm when bound to DNA to red-shifting towards 640 nm in more acidic environments [31,39]. For instance, a red-shift occurs in the acidic vesicles in granulocytes [39], the most common type of leukocyte present in urine during a UTI [40,41]. Due to this colorimetric range, the red-to-green fluorescence ratio obtained via a color imaging sensor (RG ratio) is frequently used to differentiate between cell types, as we have previously demonstrated with leukocytes [34]. Although AO has been used to stain each cell type that can be present in urine during a UTI (epithelial cells, leukocytes, and bacteria), they have been studied independently. Therefore, the unique colorimetric features of AO-stained cells could improve the contrast needed to automate the segmentation and classification of these cells that are counted in a urinalysis microscopic examination.

In this pilot study, we developed a staining and post-processing technique to test the feasibility of using AO as a contrast agent for a semi-automated urinalysis microscopic examination. We hypothesized that AO-stained cells will emit distinct colorimetric features that can be used to separate and enumerate cells in urine (leukocytes, bacteria, and epithelial cells). For this work, the fluorescence characterization and other image features of AO-stained cells were used to define classifiable properties associated with each cell type. Using that classification to enumerate the cells, the results were compared to the clinical MME results.

## 2. Materials and Methods

### 2.1. Point-of-Care (POC) Image-Based System

**Figure 1:**
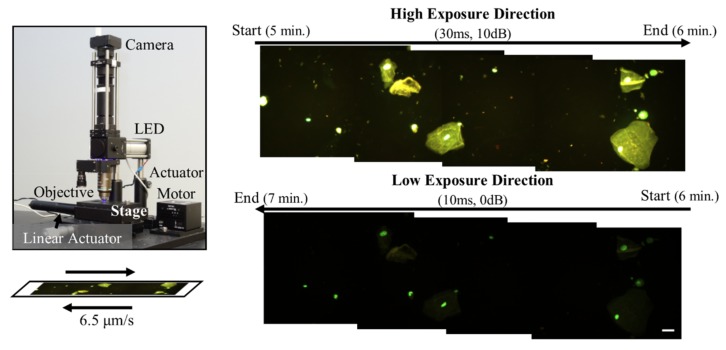
The point-of-care (POC) system (left) consisted of a Point Gray color camera, 488 nm LED, 40x Nikon objective, stage, linear actuator, and actuator motor. The diagram below the system image demonstrates the slide translation, which travels at 6.5μm/s in two directions. The camera field-of-view was not perfectly parallel to the direction of translation, therefore the image mosaics (right) were tilted. The first direction used high exposure camera settings with an exposure of 30 ms and a gain of 10 dB to image low fluorescence intensity objects (bacteria). The second direction used an exposure of 10 ms and a gain of 0 dB to image high fluorescence intensity objects (epithelial cells and leukocytes). Scale bar represents 20 μm. Images enhanced for publication.

An epi-fluorescence microscope designed to mimic features of POC optics-based systems, as previously reported in Powless, et al., was used to acquire images of urine specimens stained with AO (Figure 1) [34]. Briefly, a 488 nm LED (Philips) emitted light through a 40× Nikon Plan Fluor objective lens (NA (numerical aperture) = 0.13, Nikon Instruments Inc.), then back through the objective lens, a 475 nm dichroic filter set (Chroma Technologies, Bellows Falls, VT, USAcity, state abbreviation if available, country), and a 500 nm longpass filter (Thorlabs, Inc., Newton, NJ, USAcity, state abbreviation if available, country). The resulting image was projected via a 150 mm achromatic doublet tube lens (Thorlabs, Inc) onto a Point Grey USB 2.0 Chameleon color camera (FLIR Systems, Inc., Wilsonville, OR, USAcity, state abbreviation if available, country). The area of one field of view (FOV) was 0.15 mm^2^. Image acquisition, as shown in Figure 1, was automated using a stage setup with a linear actuator motor (Thorlabs, Inc.) and sequential image capture was performed using Point Grey software (FlyCapture SDK, Wilsonville, OR, USAcity, state abbreviation if available, country). Images were acquired at two settings (adjusting exposure and gain) to capture all cell types, since fluorescence intensity of epithelial cells and leukocytes was significantly greater than bacteria; the same FOV was imaged with the two settings. The high exposure direction had an exposure of 30 ms and gain of 10 dB. The low exposure direction had an exposure of 10 ms and gain of 0 dB. Images were acquired in two-minute increments involving both directions.

### 2.2. Human Subject Population

Study participants, suspected of a UTI, were recruited at the University of Arkansas’ Pat Walker Health Center (PWHC) under an IRB-approved study protocol (IRB #1803106650 approved April 6, 2018). The study population was 15, with 11 females and 4 males (18+ years old). Diagnoses included 3 UTIs, 7 dysuria, 2 physical pain, and 3 other minor diagnoses. All 15 subject specimens were tested with a dipstick test, 11 received a MME, and data from six subjects was successfully acquired using the POC system. Following written informed consent, urine specimens were collected by the patient mid-stream into a sterile, plastic collection cup. Samples were stored at room temperature for up to four hours and 4 °C thereafter [42].

### 2.3. Clinical Manual Microscopic Examination

The clinical MMEs were performed by trained laboratory technicians at the PWHC following a positive dipstick test. The SIEMENS Clinitek Status+ was used to read the Multistix 10 SG reagent strips (ref. 2161), and a positive test for blood, leukocyte esterase, or nitrate resulted in an MME being performed. To prepare the specimens for microscopy, a 1.5 mL microcentrifuge tube (VWR, Radnor, PA, USAmanufacture, city, state abbreviation if available, country) was filled with urine and centrifuged at 9814 RPM for 45 sec. The supernatant was decanted, and the remaining droplet was mixed to resuspend any cells on the bottom of the tube. As an optional step, Sternheimer Malbin (Astral Diagnostics 6561-01, Paulsboro, NJ, USAcity, state abbreviation if available, country), a contrast agent, was mixed with the cell sample to provide contrast for the MME. A plastic pipette was used to transfer the cell sample into a plastic cassette to form a monolayer of cells. A transmission microscope with a 40× Olympus 0.65 Plan Air objective lens (Olympus, Shinjuku, Tokyo, Japancity, state abbreviation if available, country) in phase contrast was used to examine the cell sample. Cells were manually counted in 9 FOVs (total area of 0.134 mm^2^) and averaged. Results were reported as reference ranges rather than absolute values, for instance, “Few (3–10)”.

### 2.4. Acridine Orange (AO) Staining Method

Initially, specimens were processed following the technique applied by the laboratory technicians: specimen was transferred to a microcentrifuge tube, centrifuged, and the supernatant decanted. For this study, the residual supernatant was removed in order to resuspend the cells in 50 µL acridine orange hemi(zinc chloride) salt (Sigma-Aldrich, St. Louis, MO, USA) solution at a final concentration of 100 μg/mL and pH of 7.34. Instead of using a commercial urine cassette, 8 µL of the stained sample was transferred to a glass slide and mounted with a glass coverslip (no. 1). Since AO emission fluorescence is known to red-shift over time [34], images were acquired at three incubation periods starting at 3, 5, and 7 min after applying the stain.

### 2.5. Automated Image Analysis and Cell Enumeration

The fluorescence images of AO-stained cells in urine were processed using a custom MATLAB (MathWorks, Natick, MA, USAcity, state abbreviation if available, country) algorithm to detect objects and quantify their RG ratio and area. The algorithm utilized built-in MATLAB functions to convert each image into a binary image, which enabled the use of the *regionprops* function to extract the centroid, boundaries, and area of each object within the image. Any object touching the edges of the image or with saturated pixels were excluded, as they misrepresented the actual object’s size and fluorescence, respectively. The initial classification of the AO-stained cells was performed by three laboratory technicians at the PWHC. Each technician was presented 8 full FOV images of AO-stained cells acquired by the POC system for each incubation period described in the previous section. Each object in the images was numbered and the technician rating the object as either epithelial cells, leukocytes, bacteria, or cellular debris. Following a brief introduction to fluorescence microscopy under 10 min., the technicians classified the cells based on their understanding of the cellular morphology. Since visual examinations can be subjective, three technicians participated in the classification process and interobserver agreement was evaluated [43]. The manual classification was used to define threshold ranges for each cell based on their RG ratio and area to differentiate the groups in the automated classification and enumeration algorithm. For instance, the epithelial cells area threshold range would be defined as greater than 500 μm^2^ (Supplementary Materials).

### 2.6. Performance of the Point-of-Care (POC) System Compared to the Clinical Results

Qualitatively, the fluorescence images of AO-stained cells were compared to the clinical MME, with or without the contrast agent, performed by the laboratory technicians at the PWHC. Quantitatively, the absolute cell counts determined by the POC system were compared to the clinical results which reported the count as a range of values [44].

## 3. Results

### 3.1. Qualitative Imaging Performance

**Figure 2:**
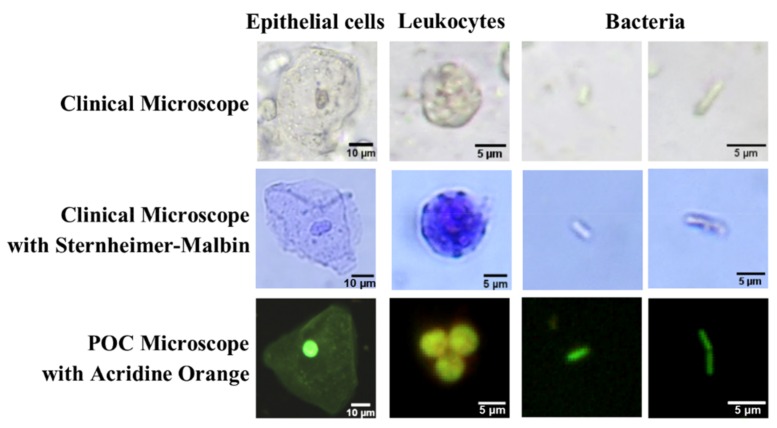
Qualitative comparison between the clinical system using phase contrast (row 1), clinical system using Sternheimer–Malbin dye (row 2), and the point-of-care (POC) system using acridine orange (row 3). Epithelial cells, leukocytes, and bacteria demonstrate the differences between the systems. Scale bars represents 10μm for epithelial cells, and 5μm for leukocytes and bacteria. Images enhanced for publication.

In Figure 2, images acquired using a clinical microscope, with and without a contrast agent, and our POC microscope with AO are compared. The Sternheimer–Malbin dye improved the visualization of the epithelial cells and leukocytes; however, bacteria were less distinct when using either clinical methods. The images acquired by the POC system exhibited greater contrast, improving the visualization of the cells. Since AO stains DNA and RNA, the nuclei in both epithelial cells and leukocytes were well-defined. For AO-stained leukocytes, the multi-lobed nuclei, indicative of granulocyte lineage, were distinguishable. For AO-stained rod-shaped bacteria, the connection between linked rods were usually clearly defined, as shown in the bottom right image in Figure 2.

### 3.2. Colorimetric and Image-Based Morphology Features

**Figure 3:**
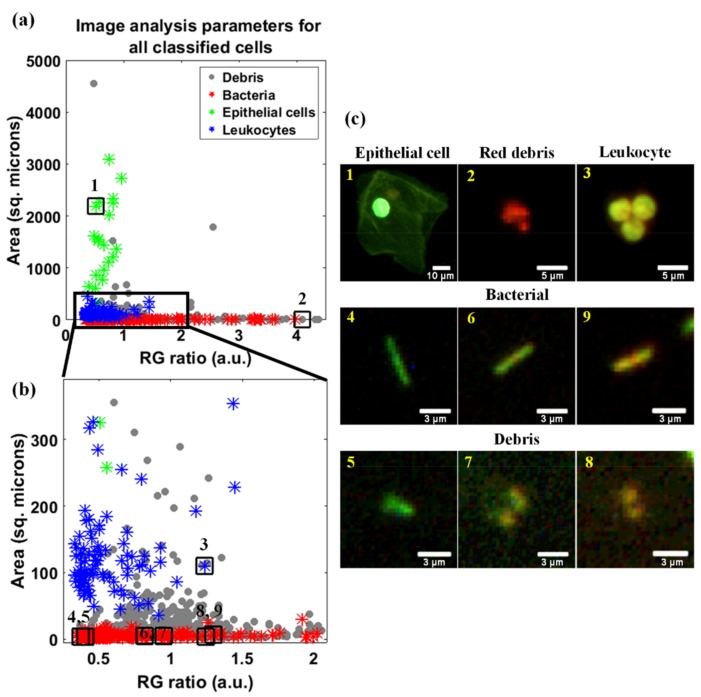
Classification performed by laboratory technicians on images acquired by the point-of-care (POC) system and the image analysis parameters (red-to-greenratio and area). (**a**) Plot of all objects detected by the POC system (*n* = 984) and their associated classification. Cellular debris is represented by gray circles, bacteria by red asterisks, epithelial cells by green asterisks, and leukocytes by blue asterisks. (**b**) A plot of the highlighted section in plot (**a**). In both plots, numbered points correspond to (**c**) images of the associated objects. Scale bars represents 10 μm for epithelial cells, 5 μm for leukocytes, and 3 μm for bacteria and cellular debris. Images enhanced for publication.

The RG ratio and area were quantified and plotted for each cell to identify threshold ranges used to differentiate and enumerate the three cell types. In Figure 3, image analysis parameters of AO-stained cells from six subjects, for all incubation periods, were plotted with assigned colors indicative of the cell types as classified by the laboratory technicians. Interobserver agreement was used to evaluate subjectivity between the technicians’ classification rating. For bacteria, epithelial cells, leukocytes, and cellular debris there was 85.8%, 93.5%, 92.4%, and 92.5% agreement, respectively. Additionally, images were included to demonstrate the RG ratio and area associated with each cell type. Using clinician-derived classification labels, there were distinct ranges associated with cell area (y-axis) that differentiated the three cell types. The epithelial cells featured an area greater than 500 µm^2^, bacteria exhibited an area less than 30 µm^2^, and leukocytes were in between those values. The technicians also classified objects as cellular debris, which are represented by gray dots in Figure 3. The majority of the cellular debris were comparable to the RG ratio and area characteristics of bacteria. The difference was in the uniformity of the individual object’s edge: cellular debris had out-of-focus edges, see Figure 3 images 5, 7, and 8. This cellular debris were automatically excluded from the final enumeration of the cells.

### 3.3. Point-of-Care (POC) Results Compare Favorably to Urinalysis Clinical Standards

Using the threshold ranges defined by the area of each cell type, the cells from six subjects were enumerated and compared to their clinical results, as reported in Table 1. The results could not be directly compared, due to the clinical results being reported as a range and the POC system results reported as an absolute value. However, the absolute values fell near or within the clinical range, except for subject 4 where epithelial cells and bacteria were under-counted.

## 4. Discussion

An MME is one component of a urinalysis used to diagnosis a UTI. This is performed by trained laboratory technicians using a brightfield or phase contrast microscope, which can be time and labor intensive. Additionally, the epithelial cells, leukocytes, and bacteria can be difficult to visualize due to poor contrast against the background, as demonstrated in Figure 2. When stained with AO, all cell types emit a range of fluorescence emission based on their cellular content. As demonstrated in Figure 2, AO improved the contrast of all three cell types and highlighted the nuclei in leukocytes, which were indistinguishable using the clinical methods. Using conventional microscopy, leukocytes are known to be difficult to differentiate from erythrocytes [10]. AO does not stain erythrocytes due to lack of nuclear content; therefore, AO would be beneficial for identifying leukocytes related to the diagnosis of UTIs [44]. AO improved the visualization of rod-shaped bacteria, however, other bacteria were more challenging to distinguish from cellular debris. Overall, after adjusting to the fluorescence images, all technicians were comfortable classifying all three cell types, except for circular bacteria.

POC automated systems aim to lower cost, improve test turnaround time, and reduce technician involvement, thus improving test accessibility. The automation of POC systems that utilize brightfield or phase contrast microscopy can be limited by poor image contrast. Fluorescent dyes can improve the contrast between the brightly stained cells and the image background allowing for improved image segmentation techniques. Specifically, AO provides enhanced contrast by its varied fluorescence emission in epithelial cells, leukocytes, and bacteria. In designing the semi-automated classification algorithm, we acquired images with two exposure settings, since cells and bacteria had different fluorescence intensities. Following contrast enhancements, bacteria were visible in the low exposure images, and therefore only those images were processed. Future acquisition of only low-exposure images will reduce the overall time to obtain the test results. Detected objects were classified as epithelial cells, leukocytes, bacteria, or cellular debris using the RG ratio and area image features, as shown in Figure 3. The RG ratio was used due to the known colorimetric features of AO-stained cells. However, for a urinalysis, the RG ratio did not contribute to the classification of the cells, which meant the red-shift in fluorescence that often occurs in AO-stained cells did not affect the test outcome. Therefore, images from all incubation periods were combined to increase cell count. This is demonstrated in Figure 3, where the area of the cells had a greater separation between groups, while the RG ratio did not show a classifiable pattern. Therefore, area was sufficient for separating the groups where epithelial cells had an area greater than 500 μm^2^, bacteria less than 30 μm^2^, and leukocytes in between. Using these threshold ranges to classify and enumerate the groups, and with the exclusion of cellular debris, the counts acquired by our system were similar to the clinical ranges.

## 5. Conclusions

In summary, this pilot study was conducted to determine the feasibility of AO as a contrast agent to improve the automation of a urinalysis MME. In evaluating the three cell types that can be present in urine during a UTI, AO-fluorescence was sufficient to segment the stained cells from the background. This allowed for automated quantification of the RG ratio and area which were used to classify and enumerate the cells. Since the RG ratio was not the contributing classification feature, there was no constraint on the AO staining method involving incubation period; therefore, the stained cells could be imaged from 3–9 min. continuously. This allowed more FOVs to be imaged, which could reduce sampling error by increase the total cell count. Limitations to the technique presented in this paper include the centrifugation of the urine specimen, which has been shown to cause error from the irregular cell resuspension [8], and the need to reduce cellular debris by emphasizing the proper clean catch method during specimen collection. Alternatively, a benefit of this system is that the images can be recorded and later reviewed by a trained technician or remotely when technicians are unavailable. Additionally, the wide range of incubation periods could potentially reduce technician training time. Overall, this semi-automated technique using AO could be a viable option for the automation of urinalysis microscopic examinations at the POC.

## Figures and Tables

**Table 1:** Comparison of the count performed by the point-of-care (POC) system to the clinical results.

		Clinical Results (Range)			POC System Results (Count)		
Subject	Diagnosis	Bacteria	Leukocytes	Epithelial Cells	Bacteria	Leukocytes	Epithelial Cells
1	Abdominal pain	0	0	Few (3–10)	0	0	0
2	Dysuria	Moderate (10–25)	5–10	Few (3–10)	30	5	1
3	Dysuria	Few (3–10)	0–5	Few (3–10)	4	0	0
4	UTI	Moderate (10–25)	5–10	Moderate (11–25)	2	13	3
5	Pelvic Pain	Moderate (10–25)	5–10	Few (3–10)	22	6	2
6	Dysuria	Trace (0–2)	5–10	Trace (0–2)	2	6	0

## Supplementary Materials

The following are available online at https://www.mdpi.com/2075-4418/9/3/122/s1. The MATLAB automated cell detection code titled “Automated image analysis of AO-stained urine specimens”. A zip file titled “Example Image Set” consisting of original images used as examples. Table S1 titled “Object data from Example image 1”.
