# Supplementary material for: Evaluation of Acridine Orange Staining for a Semi-Automated Urinalysis Microscopic Examination at the Point-of-Care
Source: Diagnostics (Basel). 2019 Sep 18;9(3):122. doi: 10.3390/diagnostics9030122 (PMC6787640; doi:10.3390/diagnostics9030122)
Supplement: Supplementary file 1 [file diagnostics-09-00122-s001.zip › Automated Algorithm.pdf]

Automated image analysis of AO-stained urine specimens

Developed by Amy Powless and assisted by Sandra Prieto 2019

Contents

- Select folder containing the images
- Find centroid and object boundaries using image segmentation

Select folder containing the images

```
%Ask user to choose folder containing the images (aka Directory)
directoryName = uigetdir;
directoryNameTIF = cat(2,directoryName,'\*.tif'); %adds wildcard.tif to Name

%Find all .tif images in directoryNameTIF
FolderImages = dir(directoryNameTIF);

addpath(directoryName);

%Count how many .tif images
NumOfIMGs = length(FolderImages);
```

Find centroid and object boundaries using image segmentation

```
ObjInCount = 1;

% Save message to confirm the code worked, but no objects were detected
complete = {'This task is complete. If there is no ObjectData variable, then this folder had 0 objects detected.'};
save('TaskCompleted','complete')


for i = 1:NumOfIMGs
    centroid=[];
    bounds=[];
    Areas=[];
    Perimeters=[];
    RG_new =[];
    MaxIntensities=[];
    Circularities=[];

    % Create name for each of the .tif files
    FolderPathName = cat(2,directoryName,'\', FolderImages(i).name);

    % Load images
    im = imread(FolderPathName);
    figure,imagesc(im);axis image;colormap gray;
    title('Original Image'), axis off;

    % Correct for uneven illumination
    % Resource: https://www.mathworks.com/help/images/correcting-nonuniform-illumination.html
    se = strel('disk',500);
    background = imopen(im,se);
    im = im - background;
    figure,imagesc(im);axis image;colormap gray; % plot flat field corrected image
    title('Illumination Corrected'), axis off;

    % Convert image to grayscale image only for segmentation
    I = rgb2gray(im);
    figure,imagesc(I);axis image;colormap gray;
    title('Grayscale'), axis off;

    % Enhance contrast
    img = imadjust(I,[0.001 .03]);
    figure,imagesc(img);axis image;colormap gray;
    title('Contrast Enhanced'),axis off;

    % Threshold to convert to binary image
    threshold = 0.5;
    imBinary = imbinarize(img,threshold);
    figure,imagesc(imBinary);axis image;colormap gray;
    title('Binary'),axis off;

    % Exclude "noise"
    minArea = 75;
    imExclude = bwareaopen(imBinary,minArea);
    figure,imagesc(imExclude);axis image;colormap gray;
    title('Excluded Noise'),axis off;

    SE = strel('disk',3);
```

```
    imageNEW = imclose(imExclude,SE);
    figure,imagesc(imageNEW);axis image;colormap gray;
    title('Final Image'),axis off;

    % Find centroids and boundary locations
    imRegion = regionprops(imageNEW,'Centroid','BoundingBox','Area','Perimeter');

    if mean(mean(imageNEW)) == 0 % Skip the following steps if no object is detect

    continue

    else

    % Compile object data for each image
    centroid = cat(1,centroid,imRegion.Centroid);
    bounds = cat(1,bounds,imRegion.BoundingBox);
    Areas=cat(1,Areas,imRegion.Area);
    Perimeters=cat(1,Perimeters,imRegion.Perimeter);

    figure,imagesc(imageNEW);axis image;colormap gray;
    hold on,plot(centroid(:,1), centroid(:,2),'r*');
    title('Final image with Detected Centroids'),axis off;

    % Crop and extract info per object in each image
    [NumOfObj,~] = size(centroid);

    mask = imageNEW;

   Counter = 1;
   C = 0;
   clear ImageData
   clear obj_info
   for b = 1:NumOfObj

       % Exclude objects touching the edges
       B1 = bounds(b,1);  % Evaluating the location in relation to the left edge
       B2 = bounds(b,2);  % Evaluating the location in relation to the top edge
       B3 = bounds(b,1)+bounds(b,3); % Evaluating the location in relation to the right edge
       B4 = bounds(b,2)+bounds(b,4); % Evaluating the location in relation to the bottom edge
           if B1 > 0.5 && B2 > 0.5 && B3 < 1296.5 && B4 < 964.5
           bounds2(b,1) = bounds(b,1);
           bounds2(b,2) = bounds(b,2);
           bounds2(b,3) = bounds(b,3)+1; % added padding for crop
           bounds2(b,4) = bounds(b,4)+1; % added padding for crop


        % Crop image tightly around each object
        C_mask = imcrop(mask,bounds2(b,:));
        [l w ~] = size(C_mask); % ~ excludes last value in 3D matrix
        C_img = imcrop(im,bounds2(b,:));
        [l w ~] = size(C_img); % ~ excludes last value in 3D matrix
        new_IMG = C_img.*uint8(C_mask); % Exclude background
        new_IMG_fig = imadjust(new_IMG,[0 0.15]); % Contrast enhance for figure


        % Calculate the red:green (RG) ratio per cropped image
        RG_img = [];
        lengthA = 0;
        widthB = 0;

        [lengthA,widthB,~]=size(new_IMG);
        for A = 1:lengthA-1;
            for B = 1:widthB-1;

            M_red = new_IMG(A,B,1);
            N_green = new_IMG(A,B,2);

            RG = double(M_red)/double(N_green);

            RG_img = cat(1,RG_img,RG);
            end
        end

RG_img(isnan(RG_img))=0; % convert NaNs to 0s
RGratio = nonzeros(RG_img);% Extract all nonzero numbers

% Average RG ratio and find max intensity of the image and circularity of the object

RG_avg = mean(RGratio);
Max_intensity = max(max(max(new_IMG)));
circularity = (4*pi*Areas(b,1))./(Perimeters(b,1).^2);

if Max_intensity < 255 % Exclude objects with oversaturated pixels
    C=C+1;
```

```
  % Store the information from each object
  L ={'Centroid X' 'Centroid Y' 'Bounds Left' 'Bounds Top' 'Bounds Width' 'Bounds Height' 'Area' 'Perimeter' 'RG ratio' 'Max Intensity' 'Circularity'};

      obj_info(C,1:2)=centroid(b,:);
      obj_info(C,3:6)=bounds2(b,:);
      obj_info(C,7)=Areas(b,:);
      obj_info(C,8)=Perimeters(b,:);
      obj_info(C,9)=RG_avg;
      obj_info(C,10)=Max_intensity;
      obj_info(C,11)=circularity;

      ALL = [L;num2cell(obj_info)];

else
end

           else
           end
   end

    % Store all object information per image
    ObjectData(ObjInCount).name=FolderImages(i).name;
    ObjectData(ObjInCount).OBJcount=length(imRegion(:,1));
    ObjectData(ObjInCount).IMG = im;

    ObjectData(ObjInCount).OBJ_info = ALL;

    ObjInCount = ObjInCount +1;

    end
end

% Save all data
save('ObjectData','ObjectData')
```

Original Image

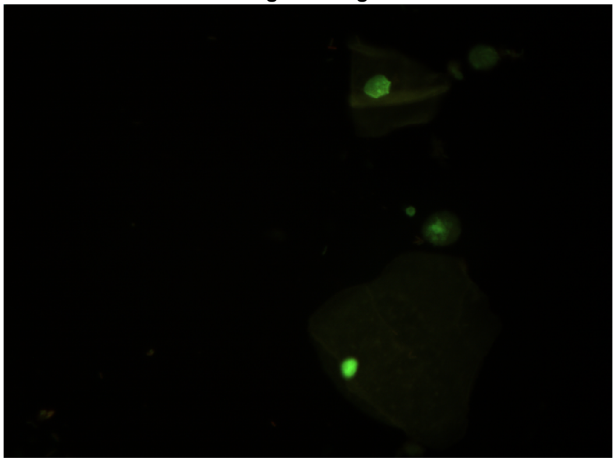

Illumination Corrected

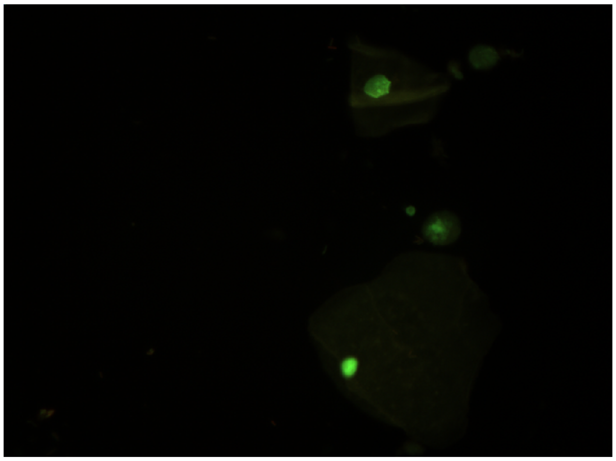

Grayscale

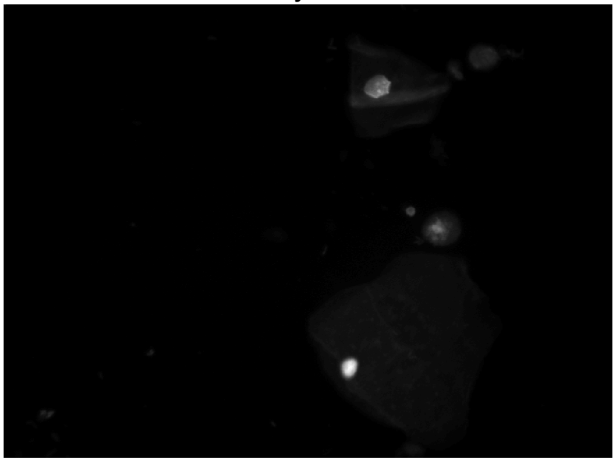

Contrast Enhanced

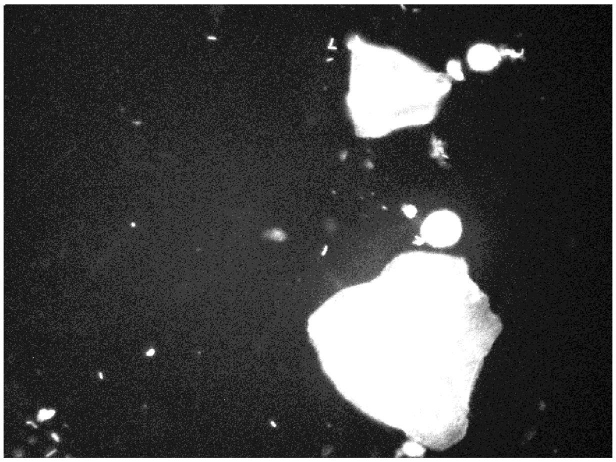

Binary

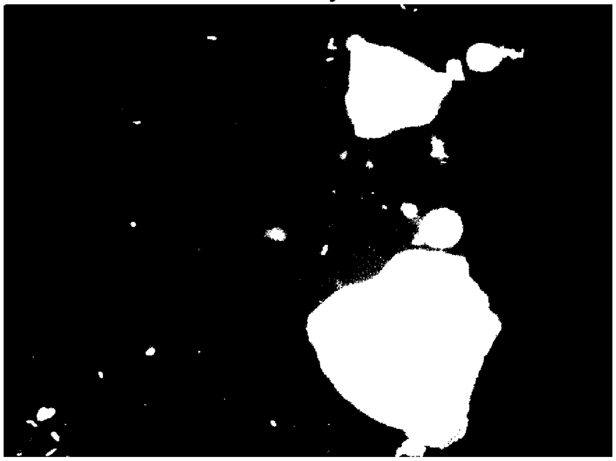

Excluded Noise

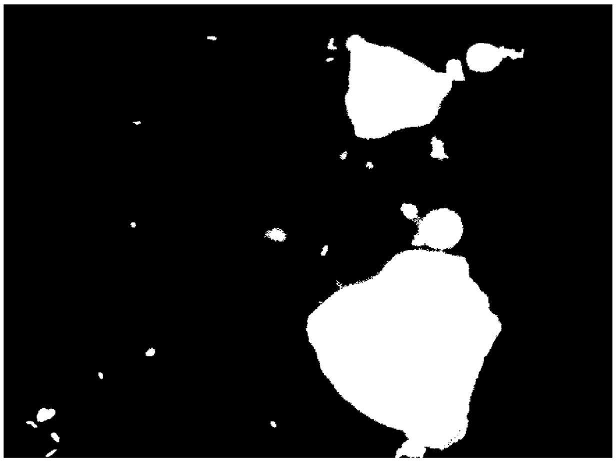

Final Image

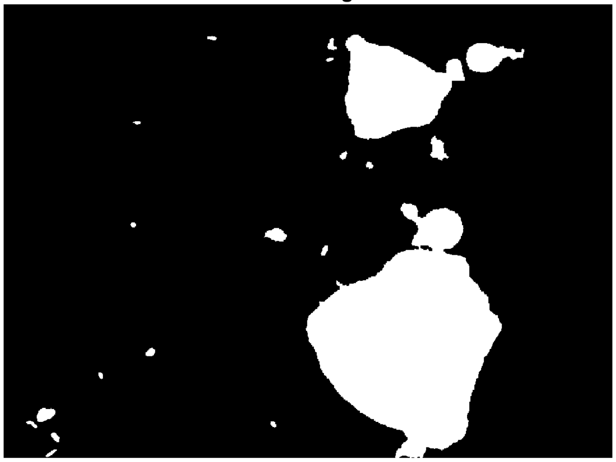

Final image with Detected Centroids

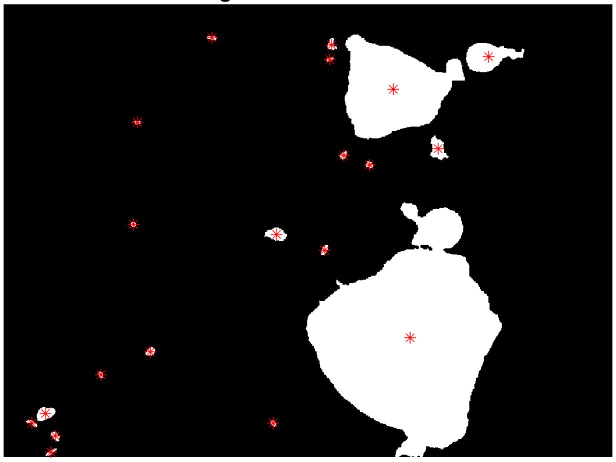

Published with MATLAB® R2017a
