# Supplementary material for: Evaluation of Acridine Orange Staining for a Semi-Automated Urinalysis Microscopic Examination at the Point-of-Care
Source: Diagnostics (Basel). 2019 Sep 18;9(3):122. doi: 10.3390/diagnostics9030122 (PMC6787640; doi:10.3390/diagnostics9030122)
Supplement: Supplementary file 1 [file diagnostics-09-00122-s001.zip › Table S1.pdf]

Evaluation of acridine orange staining for a semi-automated urinalysis microscopic examination at the point-of-care

Supplementary Material

Table S1. Object data from Example image 1 acquired using the MATLAB code titled "Automated image analysis of AO-stained urine specimens" in Supplementary Materials.

Centroid X	Centroid Y	Bounds Left	Bounds Top	Bounds Width	Bounds Height	Area	Perimeter	RG ratio	Max Intensity	Circularity
61.17	892.72	48.50	885.50	25.00	17.00	178.00	63.02	0.86	10.00	0.56
89.60	872.39	70.50	858.50	42.00	31.00	857.00	117.18	1.44	42.00	0.78
101.68	954.48	90.50	946.50	24.00	18.00	160.00	59.75	1.08	9.00	0.56
110.61	920.26	100.50	910.50	20.00	21.00	230.00	60.66	1.03	11.00	0.79
207.58	789.47	202.50	781.50	11.00	17.00	116.00	40.14	1.35	14.00	0.90
276.67	469.31	270.50	463.50	13.00	12.00	101.00	34.35	0.97	14.00	1.08
285.48	252.27	275.50	248.50	19.00	9.00	86.00	40.05	1.13	7.00	0.67
313.14	740.48	302.50	730.50	22.00	19.00	267.00	60.19	1.17	30.00	0.93
444.48	72.13	434.50	67.50	21.00	10.00	134.00	46.85	1.12	13.00	0.77
581.18	490.71	554.50	475.50	50.00	30.00	913.00	132.31	0.94	10.00	0.66
574.44	892.69	567.50	886.50	14.00	15.00	118.00	41.92	1.94	27.00	0.84
683.88	523.99	675.50	512.50	16.00	23.00	212.00	58.48	0.69	20.00	0.78
694.62	118.21	686.50	113.50	17.00	10.00	94.00	37.35	0.74	10.00	0.85
698.95	87.21	689.50	72.50	22.00	27.00	295.00	77.84	1.76	26.00	0.61
724.47	322.04	716.50	312.50	17.00	20.00	184.00	52.53	0.84	8.00	0.84
829.80	180.93	726.50	64.50	257.00	225.00	35430.00	903.45	0.82	165.00	0.55
779.70	342.46	771.50	334.50	17.00	17.00	171.00	50.16	0.67	8.00	0.85
925.21	308.61	908.50	279.50	41.00	54.00	1182.00	162.66	0.94	12.00	0.56
1033.56	111.81	984.50	82.50	125.00	63.00	4713.00	344.14	0.69	40.00	0.50
